# Neutrophil elastase (Elane) may serve as a potential therapeutic target in inflammatory bowel disease-associated growth attenuation: focus on the epiphyseal growth plate in young male rats

**DOI:** 10.3389/fendo.2025.1688220

**Published:** 2025-12-02

**Authors:** Chen Menahem, Michal Foist, Biana Shtaif, Meytal Bar-Maisels, Irit Meivar-Levy, Moshe Phillip, Galia Gat-Yablonski

**Affiliations:** 1 Gray Faculty of Medical and Health Sciences, Tel Aviv University, Tel Aviv, Israel; 2 Felsenstein Medical Research Center, Gray Faculty of Medical and Health Sciences, Petah Tikva, Israel; 3 The Laboratory for Molecular Endocrinology and Diabetes, The Institute for Endocrinology and Diabetes, Schneider Children’s Medical Center, Petach Tikva, Israel

**Keywords:** growth plate, DSS, IBD, ELANE, inflammation, Dextran sodium sulfate

## Abstract

**Introduction:**

Growth is frequently compromised in children with inflammatory bowel disease (IBD). With the prevalence of pediatric IBD having doubled over the past 25 years, growth failure poses a major additional challenge in managing the disease. Despite the use of conventional treatments: growth hormone, glucocorticoids, anti-inflammatory cytokine therapies, and bowel resection—growth impairment often persists, and some therapies may even worsen the condition. A deeper understanding of the underlying mechanisms affecting the epiphyseal growth plate (EGP) is essential for developing new therapies to address IBD-related growth attenuation. In the current study we investigated the mechanisms by which dextran sulfate sodium (DSS)-induced colitis impairs growth at the EGP. DSS is a widely utilized model for investigating the pathophysiology of IBD, however, the effect on linear growth was not reported.

**Methods:**

Young male SD rats were treated with 1% DSS in the drinking water. Body weight, colon and humerus length, and EGP height were measured. Total RNA extracted from the EGPs was used for RNA sequencing. ATDC5 cell culture was used to confirm the results.

**Results:**

Exposure to DSS led to a marked decrease in both body weight (by 20%) and colon length (by 30%). The height of the EGP was reduced by 23%, with comparable reductions in both the proliferative and hypertrophic zones. Transcriptomic analysis revealed an upregulation of immune-related genes, with neutrophil elastase (ELANE) being particularly prominent. Gene set enrichment analysis indicated downregulation of oxidative phosphorylation pathways and upregulation of cell cycle pathways, pointing to disruptions in core cellular processes such as replication and metabolism within EGP cells. *In vitro* studies supported the RNA sequencing results, showing that ELANE expression is inducible by inflammatory stimuli.

**Discussion:**

These findings suggest that immune-related genes are actively expressed in the EGP in response to systemic inflammation. Importantly, ELANE was found to be endogenously expressed in chondrocytes and upregulated under inflammatory conditions, indicating a potential intrinsic chondrocyte-mediated mechanism contributing to inflammation-induced growth attenuation. Our study introduces a novel mechanism through which chronic inflammation may directly hinder skeletal growth. This intrinsic ELANE expression, together with the upregulation of MMP13, indicates a direct catabolic impact on the extracellular matrix.

## 1 Introduction

Growth is often impaired in children with chronic inflammatory bowel disease (IBD), particularly in Crohn’s disease (CD), where up to 56% of affected children experience growth retardation (1). With the overall prevalence of pediatric IBD having doubled over the past 25 years, growth failure represents a significant additional challenge in disease management, with profound effects on both the quality of life and psychological well-being of affected children. Malabsorption, decreased nutritional intake and increased energy requirements alongside inflammation in the bowel, lead to growth attenuation and final short stature. As intervention in linear growth is efficient only during childhood, before puberty begins, it is of paramount importance to add linear growth to the therapeutic consideration in children and adolescents with IBD and support it accordingly. In many cases, in children with IBD standard growth hormone (GH) therapy has limited efficacy in promoting linear growth, likely due to GH insensitivity, a phenomenon also observed in malnourished children (2). Conventional treatments, including glucocorticoids (3), anti-inflammatory cytokine therapies (3), and bowel resection (4) have not resolved this growth impairment, and some interventions may even exacerbate it (3, 4).

Linear growth is driven by the sequential replacement of chondrocytes within the cartilaginous growth center of long bones, the epiphyseal growth plate (EGP), located at both ends of long bones, by osteoblasts (5). This process is tightly regulated by intricate interactions among systemic hormones, local growth factors, and components of the extracellular matrix (ECM). Endochondral ossification begins with early chondrocyte proliferation in the reserve zone (RZ), followed by alignment of the cells in columns in the proliferation zone (PZ), and culminates in maturation to hypertrophic chondrocytes (HZ) (6). The hypertrophic cells then cease dividing, expand in size significantly, and enhance ECM deposition and matrix vesicle secretion for mineralization. They then either undergo programmed cell death, trans-differentiation to osteoblasts (6) or autophagy (6), leading to ECM calcification and the replacement of cartilage with bone (7).

**Figure 1:**
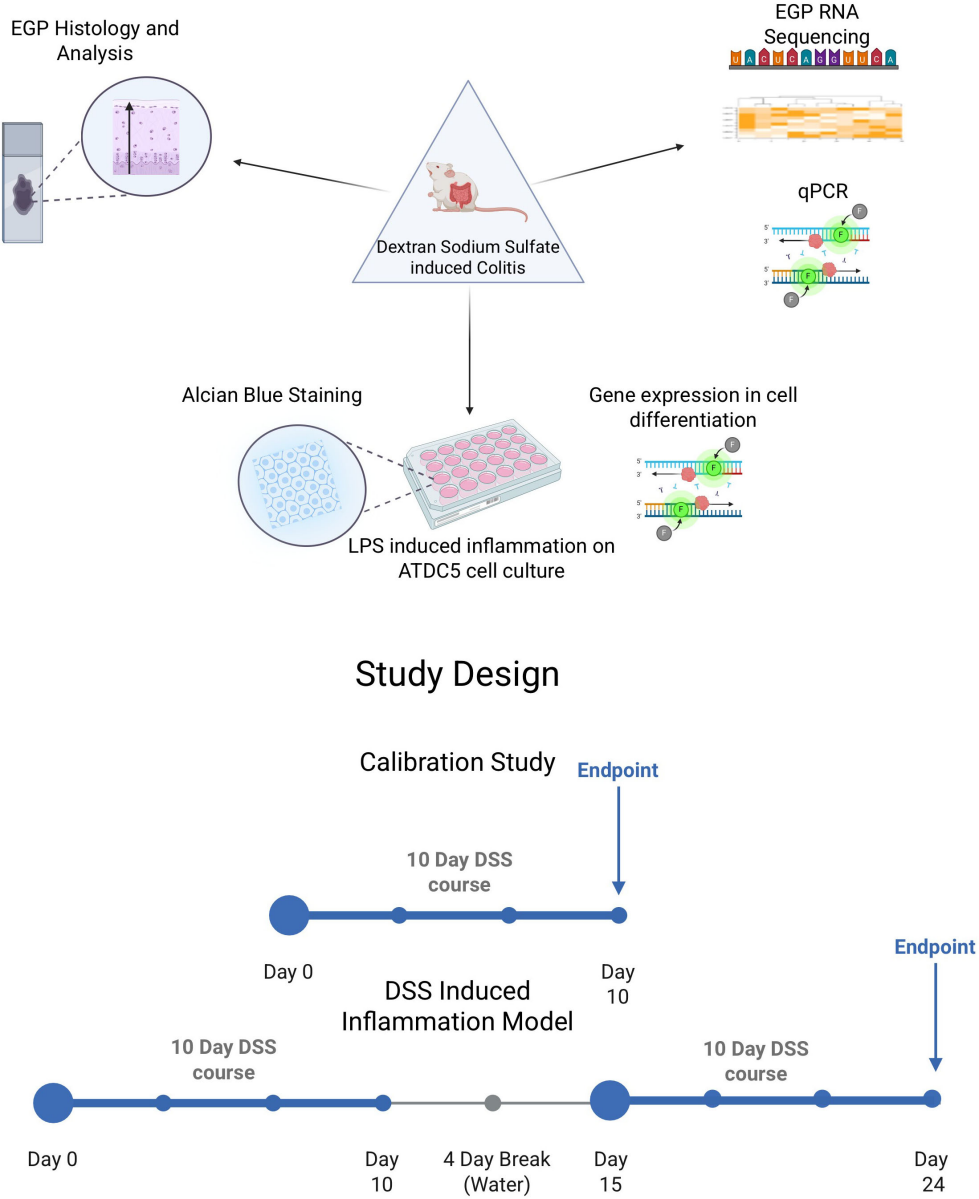
Graphical abstract and study design (Biorender, https://app.biorender.com/).

In the current study, we investigated the effect of dextran sulfate sodium (DSS) -induced colitis on bone elongation in young male rats (8, 9). Administration of DSS in drinking water leads to disruption of the intestinal epithelial barrier, resulting in mucosal inflammation, immune cell infiltration, and ulceration—features that closely resemble human IBD. DSS is a widely utilized model for investigating the pathophysiology of inflammatory bowel disease (IBD) (8–11) however, the effect on linear growth was not reported. In the current study, we focused on the impact of DSS treatment on the EGP, as a deeper understanding of the intrinsic mechanisms within the EGP that influence growth during IBD is essential for developing new therapies to counteract IBD-induced growth attenuation (**Figure 1** – Graphical Abstract).

## 2 Materials and methods

### 2.1 Animals

In all the experiments, we used young (21-24d) male Sprague Dawley rats (purchased from Envigo Laboratories Ltd., Jerusalem, Israel). The experiments were conducted at the animal care facility of the Felsenstein Medical Research Center (FMRC), according to the guidelines of the Institutional Animal Care and Use Committee of Tel Aviv University to which the FMRC is affiliated. The approval was obtained before the experiments were initiated (Approval number 01-20-088). During the acclimation, dosing and recovery phases, the animals were housed in conventional, polycarbonate cages with solid bottoms (3 per cage) with a plastic cylinder for shelter and comfort. Standard procedures and conditions were applied for animal care, feeding, and maintenance of room, caging, and the environment. The animals were fed the commercial 2018SC diet (Envigo Laboratories Ltd., Jerusalem, Israel) ad libitum, with free access to tap water. Automatically controlled environmental conditions were set to maintain a temperature at 21^0^C + 2^0^C with 50% + 5% relative humidity, 12:12hr light: dark cycle, and lights off at 19:00h. Throughout this study, the animals were observed daily for changes in skin, fur, and behavior in response to handling. All remained within the normal limits for this model.

### 2.2 Dextran Sodium Sulfate treatment

There were two experimental setups: A pilot study using 1% and 1.5% DSS was conducted to determine appropriate dosage for the remainder of the study and consisted of one cycle of 10 days DSS treatment. For the long study DSS, was administered twice- 10 days followed by a 4 day break, and then an additional 10 day course. All rats were sacrificed at a single time point, at the end of the study. Following the pilot study to determine the appropriate dose of DSS, a chronic colitis model was established by administering two consecutive courses of DSS with a break in between. Rats were randomized into 2 groups (CTL group n=6; DSS group n=10). The DSS group received DSS (1%) in their drinking water for 10 days, followed by a 4-day recovery period with regular water, and then a second 10-day course of DSS treatment.

### 2.3 Growth and disease monitoring

Food consumption and weight gain were measured throughout each experiment. All of the rats were euthanized by CO2 inhalation at the end of the experiments. At sacrifice, blood and spleen were collected and frozen for later analysis. Colons were removed (cecum to rectum) and measured to assess disease severity. The humeri of the euthanized animals were carefully removed, cleaned, measured for length with a digital caliper and fixed immediately for histo-morphometric analysis. EGP from the tibia was sterilely scraped using a clean dental pick dipped in 3% hydrogen peroxide and immediately flash-frozen in liquid nitrogen for subsequent analysis.

### 2.4 Measurement of EGP height

The humeri were fixed in 4% neutral buffered formalin for 48h at room temperature, decalcified with Surgipath Decalcifier II (Leica Biosystems Richmond, Inc. USA) for several hours, dehydrated through a graded ethanol series (70%, 95%, and 100%), and stabilized by two sequential changes of chloroform for paraffin embedding. EGP height measurements were performed on deparaffinized 6μm thick sections stained with hematoxylin-eosin (H&E) and Alcian blue (AB). For each bone, two sections were measured, for a total of five measurements per section. The height of the EGP was measured by drawing a straight line from the apical border of the reserve zone cells to the lower border of the mineralized cartilage. The PZ and HZ were measured separately from the apical border to the first hypertrophic cell and then from there to the lower border to the EGP, respectively. The slides were photographed under an Olympus BX40 microscope equipped with an Olympus DP71 camera (Olympus Optical Co. GmbH, Hamburg, Germany) and analyzed with Image-Pro software (version 4.5.1.22, Media Cybernetics, Inc., Rockville, MD, USA) by two specialists blinded to their origin. The findings presented in the results section represent the average of five measurements in each section.

### 2.5 RNA extraction, evaluation, sequencing and expression analysis

RNA was extracted from both EGPs and spleens using the RNeasy Mini Kit (Qiagen, Valencia, CA, USA). For EGP samples, tissues were initially minced on ice with a sterile scalpel, then further homogenized using a pellet crusher and subsequently passed through a 21G needle and syringe. Spleens were homogenized solely with a pellet crusher. RNA precipitation was carried out using 100% ethanol pre-cooled to -80 °C. RNA quality and integrity were assessed with the Agilent 2200 TapeStation system, and all samples demonstrated RNA integrity numbers (RIN) ranging from 7.6 to 9.4. RNA extraction from ATDC5 cells - RNA was isolated using TRI reagent (Sigma Aldrich, cat. No. T9424), and Chloroform collecting the aqueous phase after centrifugation (4°C, 15 min. 14,000 rpm). RNA was precipitated for 5 min with isopropanol in RT, and pellet by centrifugation (4°C, 8 min. 12,000 rpm.) RNA pellets were washed in 70% ethanol, centrifuged (4°C, 5 min. 7500 rpm) and dried. RNA was dissolved in DNase/RNase-free pure water. RNA quantity and purity were determined by NanoDrop™ One Spectrophotometer (Thermo Scientific).

Sequencing Libraries were prepared by the Grand Israel National Center for Personalized Medicine (G-INCPM) at the Weizmann Institute of Science and full mRNA sequencing was performed. Single end reads were sequenced on 2 lanes of an Illumina NovaSeq/NextSeq. The output was ~34 million reads per sample.

### 2.6 Bioinformatics analysis

Raw RNA sequencing reads were processed at the Bioinformatics Unit of the Weizmann Institute of Science. Libraries were sequenced on an Illumina NextSeq 500 platform, generating 75 bp single-end reads. Adapter sequences and poly-A/T stretches were removed using Cutadapt (12), and reads shorter than 30 bp were discarded to eliminate low-quality or uninformative fragments. Reads were aligned to the rat reference genome (Rnor_6.0) using STAR (13) with gene annotations from Ensembl release 105. The alignment was performed using the alignEndsType EndToEnd option to prevent soft clipping, and outFilterMismatchNoverLmax 0.04 was set to filter out alignments with more than 4% mismatches relative to read length. To remove PCR duplicates, Picard MarkDuplicates was used with the BARCODE_TAG parameter enabled to collapse reads sharing the same UMI (unique molecular identifier). Gene-level quantification was performed with HTSeq-count (HTSeq. (n.d.). Retrieved from http://www-huber.embl.de/users/anders/HTSeq/doc/overview.html ), using the intersection-strict mode and the same Ensembl GTF annotation file. Differential expression analysis was conducted using DESeq2 (14) within the R v4.2.2 environment. The parameters betaPrior=FALSE, cooksCutoff=FALSE, and independentFiltering=FALSE were applied to allow full dispersion modelling without fold-change shrinkage, retain all genes including those with outlier counts or low expression, and ensure that no independent filtering excluded low-abundance genes from the results. P-values were adjusted for multiple testing using the Benjamini-Hochberg method. All computational steps were managed and executed via a Snakemake workflow (15) to ensure reproducibility.

### 2.7 ATDC5 cell culture

The chondrogenic ATDC5 cell line (16) was cultured in Dulbecco’s Modified Eagle Medium/Ham’s-F12 (1:1 mixture, Gibco/Life Technologies, Thermo-Fisher Scientific) containing 5% heat-inactivated fetal calf serum, 1% l-glutamine and 1% penicillin/streptomycin (Sartorius, Israel) at 37 °C in a humidified atmosphere of 5% CO_2_ in air. Cells were seeded at an initial density of 12 × 10^3^ cells/cm^2^ in a 24-multiwell plate (Corning, USA) as previously reported (17). The next day, the cells were induced to differentiate by the addition of insulin, transferrin and sodium selenite (ITS) (Sigma-Aldrich, Israel) for 7, 14 or 21 days to obtain cells at different stages of differentiation. In the LPS experiment ATDC5 cells were incubated with or without 100 ng/ml lipopolysaccharide (LPS) during every medium change following the initial seeding and harvested at days 7, 14 and 21.

### 2.8 Quantitative real‐time PCR Relative quantification real-time PCR (qPCR)

Relative quantification real-time PCR (qPCR) relative quantification real‐time PCR was performed with Fast SYBR^®^ Green Master Mix (Thermo Fisher Scientific) according to the manufacturer’s protocols, 2μL of cDNA template, and the gene specific primer sets (**Table 1**). qPCR was carried out in the StepOnePlus™ Real-Time PCR System (Applied Biosystems). Relative gene expression was calculated using the 2^–ΔΔCT^ method and GAPDH was used as a housekeeping gene for normalization.

**Table 1:** List of primers used in qPCR.

Gene Name	Forward Primer	Reverse Primer
ELANE	AGCTCAATGCTCAGCTACC	GCCACACACGGAGTCTGTTA
Ctsg	CTGCAGCTGAGGAGTAGAGC	AGTACATTTGTCCCCCTGCG
TLR4	GCTGGGACTCTGATCATGGC	TCTGATCCATGCATTGGTAGG
IL1β	GGAGAACCAAGCAACGACAAAATA	TGGGAACTCTGCAGACTCAAAC
IL6	ATGGATGCTACCAAACTGGAT	TGAAGGACTCTGGCTTTGTCT
ColX	ACCCTGGTTCATGGGATGTTT	TATTGTGTCTTGGGGCTAGCAA
MMP13	ATGAAGACCCCAACCCTAAGC	ATGGCATCAAGGGATAGGGC
GAPDH	TGATGGGTGTGAACCACGAGA	TGGTCATGAGCCCTTCCACAA

### 2.9 Statistical analysis

T-test was used to test for significant differences between the groups in experiments that contained two groups. The significance level was set at p<0.05. One way ANOVA was used in experiments containing more than two groups. The significance level was set at p<0.05.

## 3 Results

### 3.1 Pilot study to determine an effective and safe DSS dose

**Figure 2:**
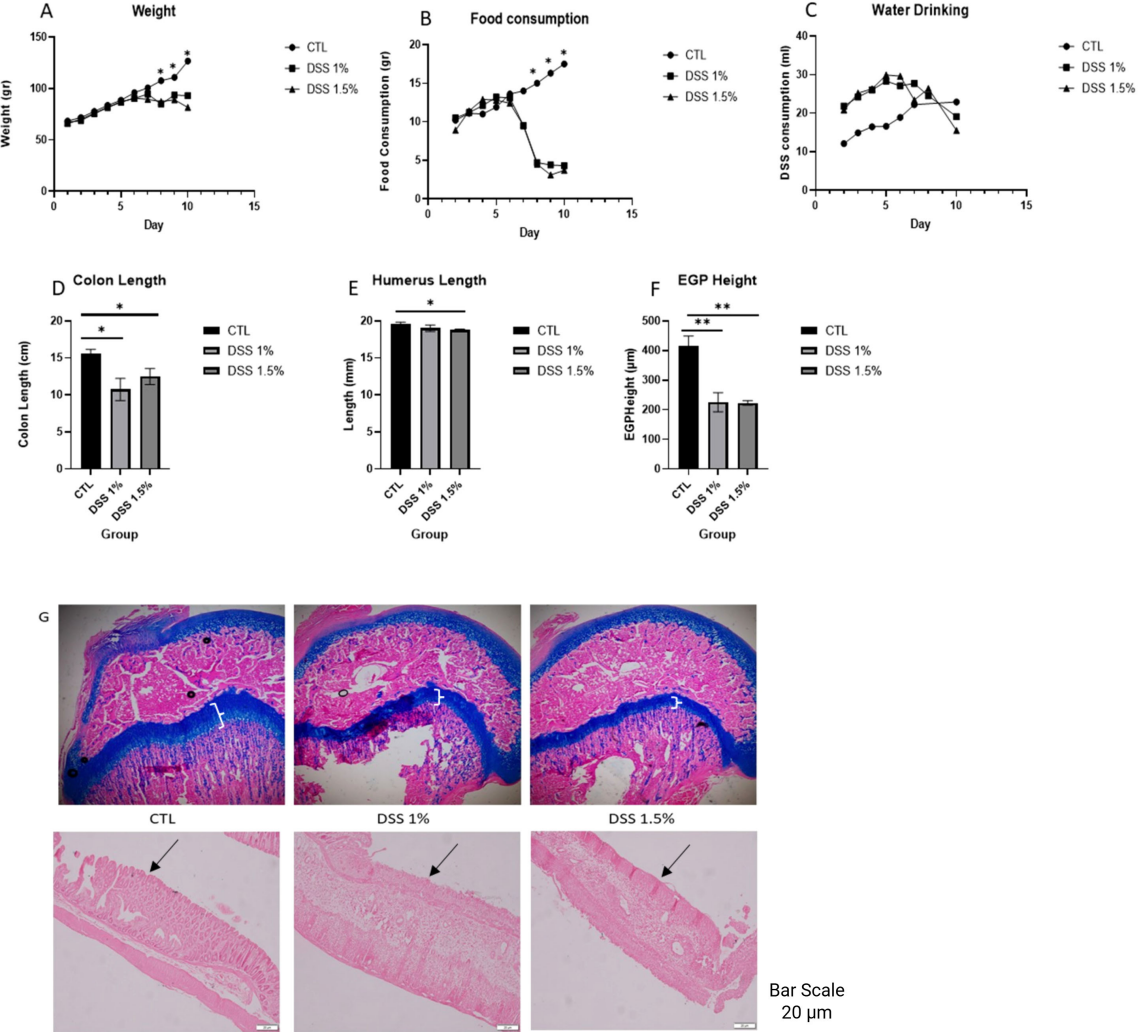
The effect of DSS (1% and 1.5%) on **(A)** weight), **(B)** food consumption, **(C)** DSS and water drinking, **(D)** Colon Length, **(E)** Humerus Length, **(F)** EGP Height, **(G)** Representative stained sections of EGP and distal rectum from all groups. Mean± SE values are shown (n=3 per group). White parenthesis showing EGP area, black arrows point to villi in distal colon. * p<0.05, **p<0.01.

The standard DSS concentration commonly used to induce colitis in adult rats [(3%–5% DSS) in drinking water] proved fatal for our juvenile rats, with complete mortality observed within six days (data not shown). Consequently, we aimed to identify a DSS concentration that could reliably induce colitis without compromising survival. A 10-day pilot study was conducted using lower DSS concentrations (1% and 1.5%). Rats were randomized into three groups: control (CTL, n=3), 1% DSS (n=4), and 1.5% DSS (n=4). DSS was provided via drinking water for 10 days in the treatment groups. Body weight, food consumption, and water drinking (as an indirect measure of DSS intake) were recorded throughout the study, and significant differences were noted in both weight and food consumption in the final days of the DSS course (**Figure 2A**). At sacrifice, colon length was significantly reduced in both DSS-treated groups compared to controls (p<0.05; **Figure 2**). Humerus length was significantly shorter in the 1.5% DSS group compared to controls (p<0.05; **Figure 2**), and EGP height was markedly decreased in both DSS-treated groups, showing a ~50% reduction relative to controls (p<0.01; **Figure 2**). Representative AB and H&E staining of EGPs and distal colon sections show effect of inflammation on EGP height and colon tissue, where EGP was shown to be significantly shorter in DSS group and thinning of the colon mucosa was observed (**Figure 2G**).

### 3.2 The effect of DSS induced inflammation on EGP height and organization

**Figure 3:**
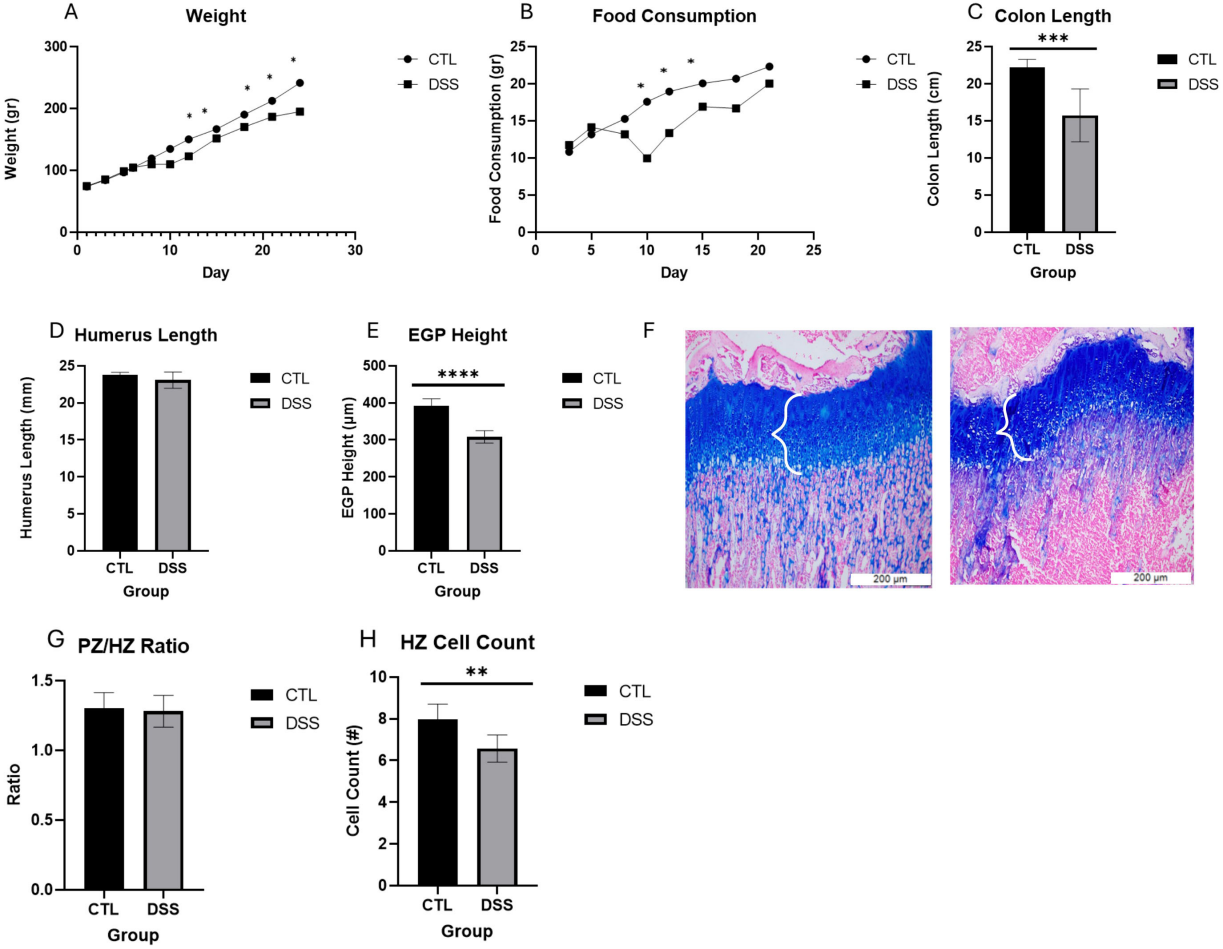
The effect of chronic DSS induced colitis on **(A)** Weight), **(B)** Food Consumption, **(C)** Colon Length, **(D)** Humerus Length, **(E)** EGP Height, **(F)** Representative stained sections of EGP- Left- CTL, Right-DSS, **(G)** PZ/HZ ratio, **(H)** HZ cell count. Mean± SE values are shown (n=6 CTL and n=7 DSS per group). **p<0.01, *** p<0.001, **** p<0001.

Since the effect on EGP height was the same in both the 1% and 1.5% DSS treated groups after 10 days, we continued our studies with the lower dosage. In the following experiments there were two courses of DSS and a 4-day interval of water only, in accordance with the established model for IBD^10,11^. Throughout the experiment from day 7, until the end, DSS treated rats weighed less than the CTL group (by 20%, p<0.05, **Figure 3A**). A significant reduction in food consumption was observed during the first course of DSS treatment (days 5–10), whereas during the recovery period and the second DSS course, the differences persisted as a non-significant trend. Notably, DSS-treated rats consumed more food during the second course compared to the first (**Figure 3B**). Colon length was measured at sacrifice and a significant reduction was noted in the DSS group (by 30%, p<0.001, **Figure 3**.). In DSS-treated rats humerus length showed a tendency to be shorter (by 3.5%, *p* = 0.05; **Figure 3**), and EGP height was significantly reduced (by 23%, p<0.0001, **Figure 3E, F**). Analysis of the EGP’s internal organization revealed that both the proliferative zone (PZ) and the hypertrophic zone (HZ) were significantly shorter in DSS-treated rats, respectively. However, the ratio between the PZ and HZ (PZ/HZ) remained unchanged indicating a proportionate reduction of both areas. (**Figure 3G**. The DSS-treated group had fewer cells in each hypertrophic column compared to the CTL group (by approximately 20%, p<0.01 **Figure 3H**), while the average calculated cell size in the EGP was the same for both the CTL and DSS-treated rats.

### 3.3 RNA Seq results

**Figure 4:**
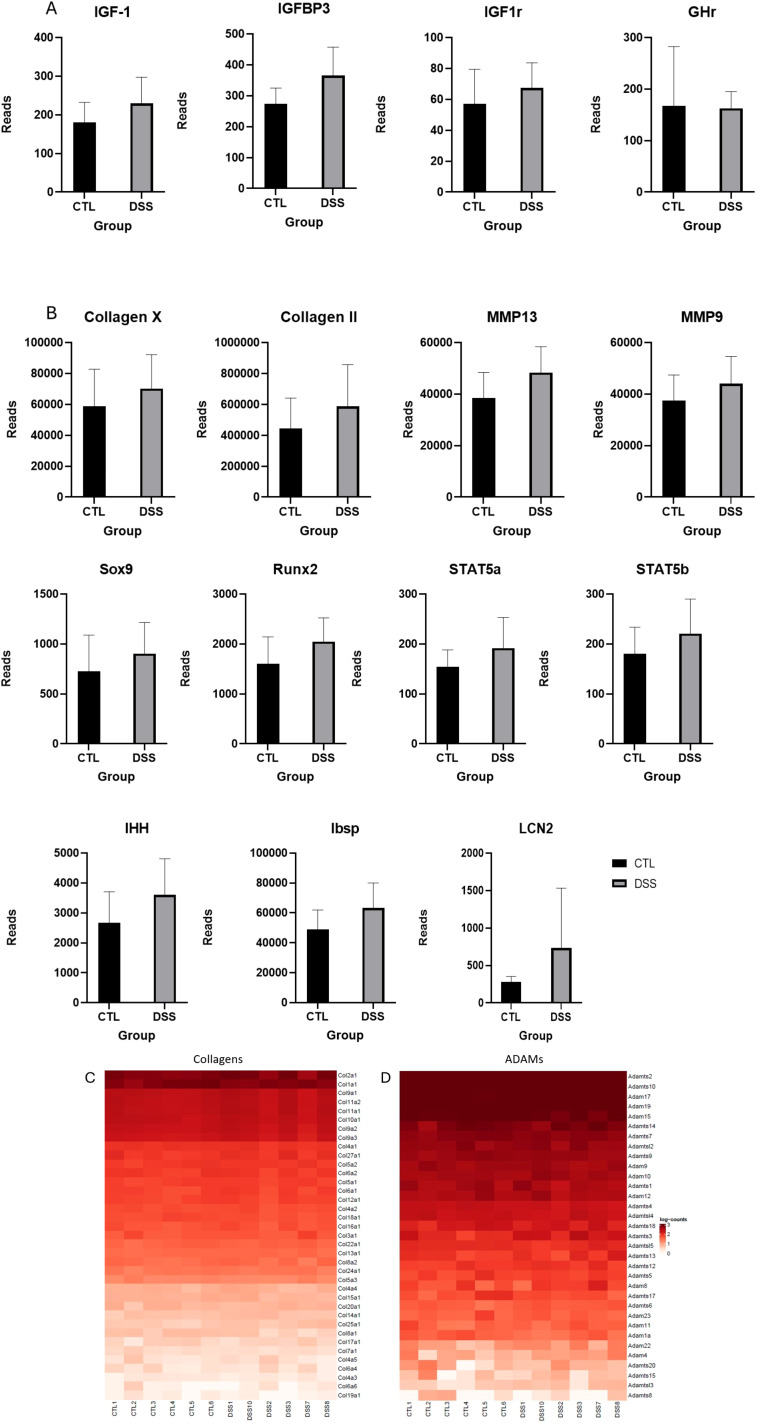
The effect of chronic DSS induced colitis on **(A)** the GH-IGF1 axis **(B)** EGP differentiation factors, **(C)** Collagens and **(D)** ADAMs. Results presented as normalized reads between groups.

**Figure 5:**
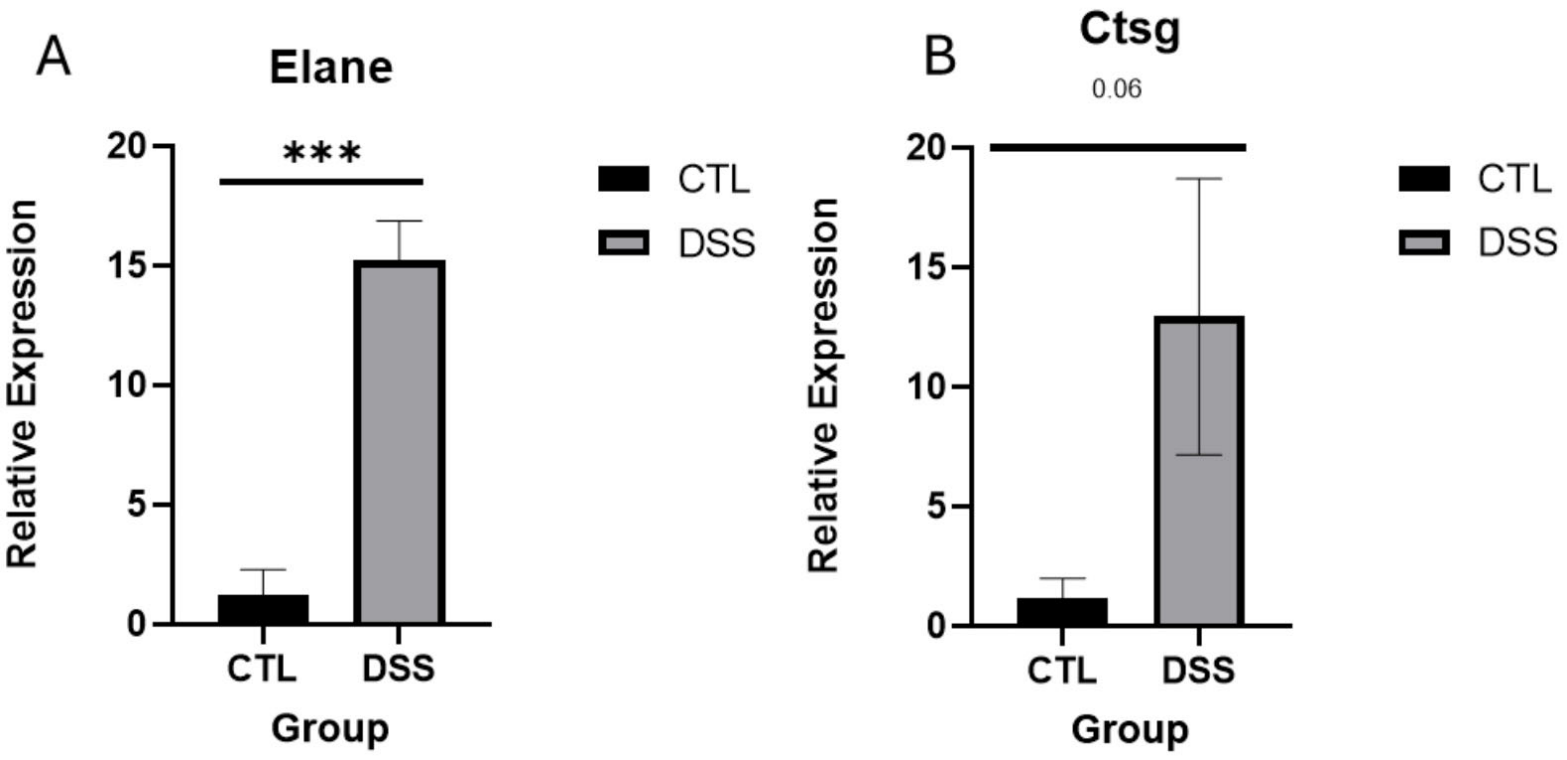
qPCR validation of RNA Seq DE genes **(A)** ELANE, **(B)** Ctsg ***p< 0.001.

To investigate the molecular impact of inflammation on the EGP, RNA was extracted from the EGPs of both control and DSS-treated groups and subjected to RNA sequencing. Initial analysis focused on genes associated with growth and development. Many genes typically expressed in the EGP were detected at high levels. Despite the pronounced shortening of the EGP height, their expression did not differ significantly between the control and DSS groups (**Figure 4**). Notably, the differentially expressed (DE) genes were predominantly immune-related, as detailed in **Table 2**; the most significant were Neutrophil elastase (ELANE), myeloperoxidase (MPO) and Catepsin G (Ctsg), all are known to be leukocyte-derived serine proteinases. qPCR results validated ELANE and Ctsg (pv=0.06) expression (**Figure 5**). STRING (https://string-db.org/) analysis of all DE genes showed strong connections between these 3 genes, which prompted further research on their role in the inflammation induced EGP shrinkage (**Supplementary Figure 1**).

**Table 2:** Initial bioinformatic analysis of EGPs sequenced.

Gene name	P. value	P. adj	Log2foldchange
Ctsg (Catepsin G)	1.83E-05	0.064	4.9
MPO (Myeloperoxidase)	6.93E-05	0.079	4.41
Elane (Neutrophil Elastase)	0.000117	0.096	4.39

Gene names as presented in raw data, p-value for each individual gene before adjustment for multiple comparisons, p. value adjusted for multiple comparisons, Log2 of fold change from reads.

### 3.4 Bioinformatics analysis

**Figure 6:**
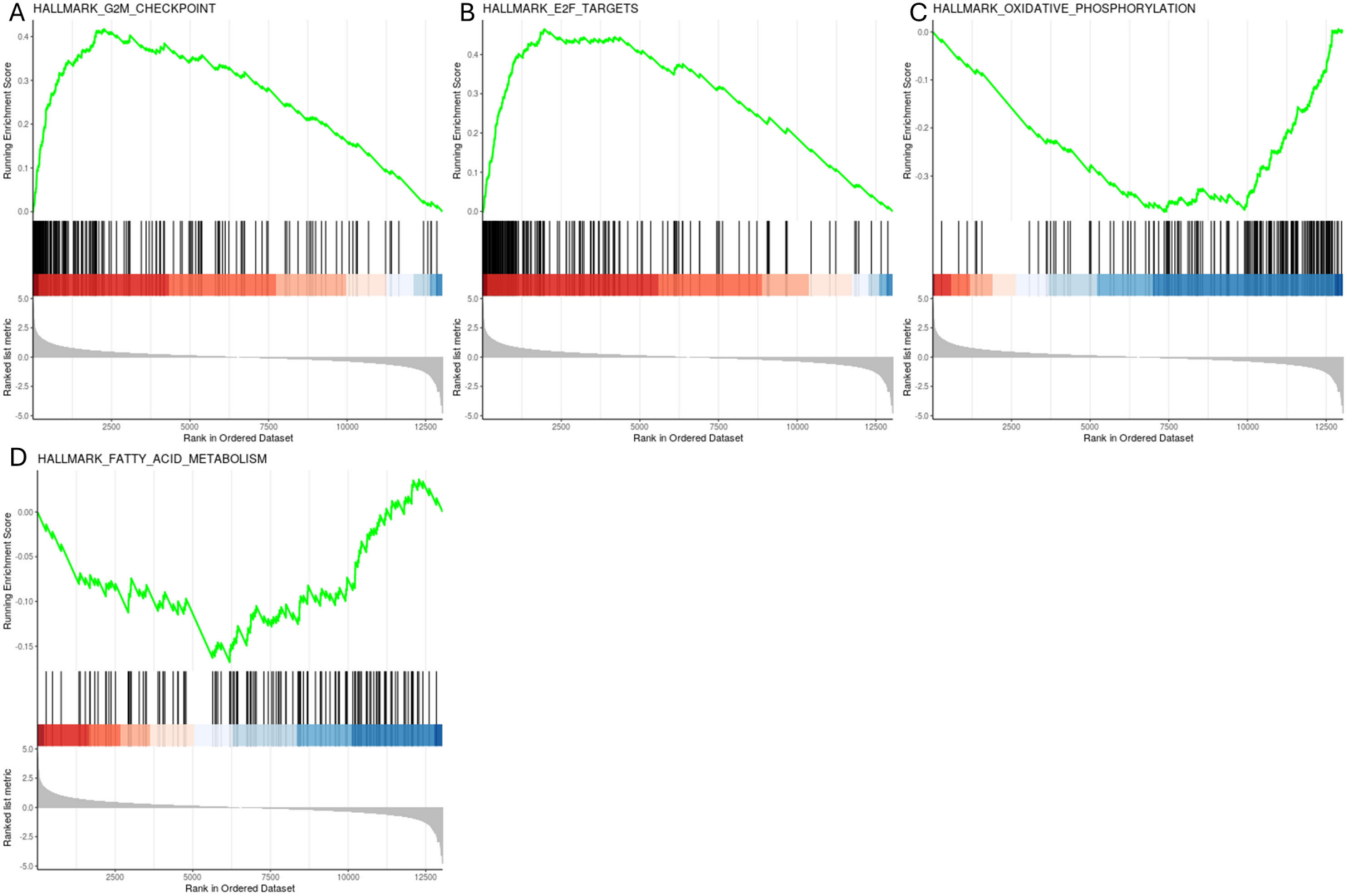
GSEA plot analysis for enriched data sets **(A)** G2/M checkpoint, **(B)** E2F Targets, **(C)** Oxidative Phosphorylation, **(D)** Fatty Acid Metabolism.

**Figure 7:**
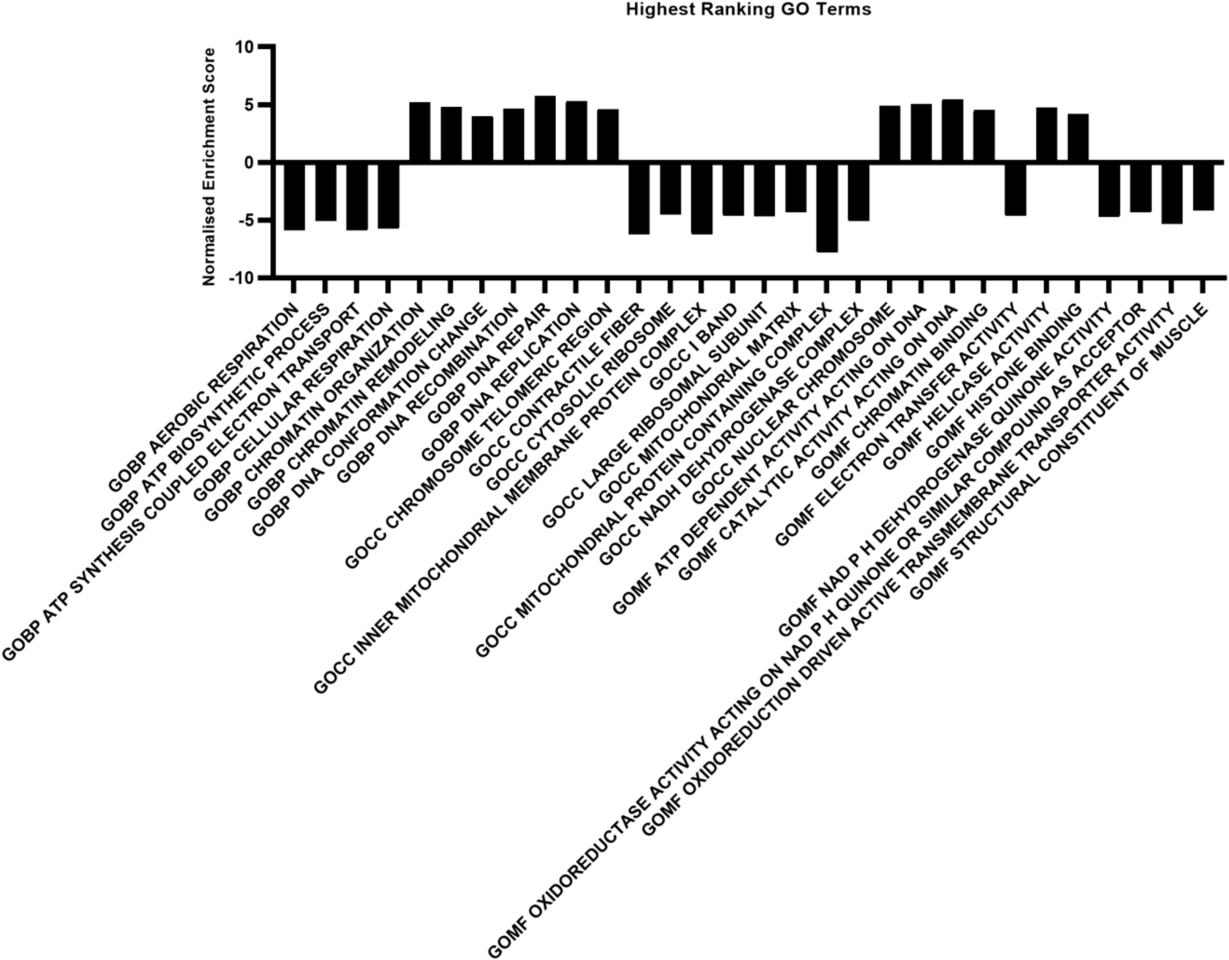
30 Highest ranking GO terms for enriched pathways in EGPs. All terms were significantly enriched. p<0.01.

To obtain a broader and more integrated understanding of pathway-level alterations within the EGP, Gene Set Enrichment Analysis (GSEA) was performed on RNA seq data. This analysis revealed changes in basic metabolic pathways, including down regulation of nutrient uptake (fatty acid metabolism and oxidative phosphorylation) and up regulation of cell cycle arrest related pathways (the G2M Checkpoint signaling pathway and E2F targets) (**Figure 6**). Gene Ontology (GO) analysis supported the transcriptional stress response observed in DSS-treated EGPs. Among the significantly enriched biological processes were DNA repair, double-strand break repair, regulation of DNA metabolic processes, and chromosome organization, suggesting activation of pathways involved in maintaining genomic integrity (**Figure 7**).

We next analyzed the DE genes by clustering them into relevant biological pathways using DAVID (Database for Annotation, Visualization and Integrated Discovery; https://david.ncifcrf.gov/). This analysis revealed that many immune related pathways were enriched. The most notable were “response to LPS”, “innate immune response” and “neutrophil extracellular trap formation” (**Supplementary Figure 2**). Two possible explanations may account for these findings: DSS may have triggered immune cell infiltration into the EGP, particularly neutrophils, which express ELANE, MPO, and Ctsg during inflammation. Alternatively, EGP chondrocytes themselves may have upregulated these genes in response to DSS. To assess the possible infiltration of immune cells into the EGP, immunofluorescent assays were conducted using anti CD45 antibody (CD45 is an immune marker present on all hematopoietic cells that are not red blood cells) with no avail.

### 3.5 *In vitro* studies

**Figure 8:**
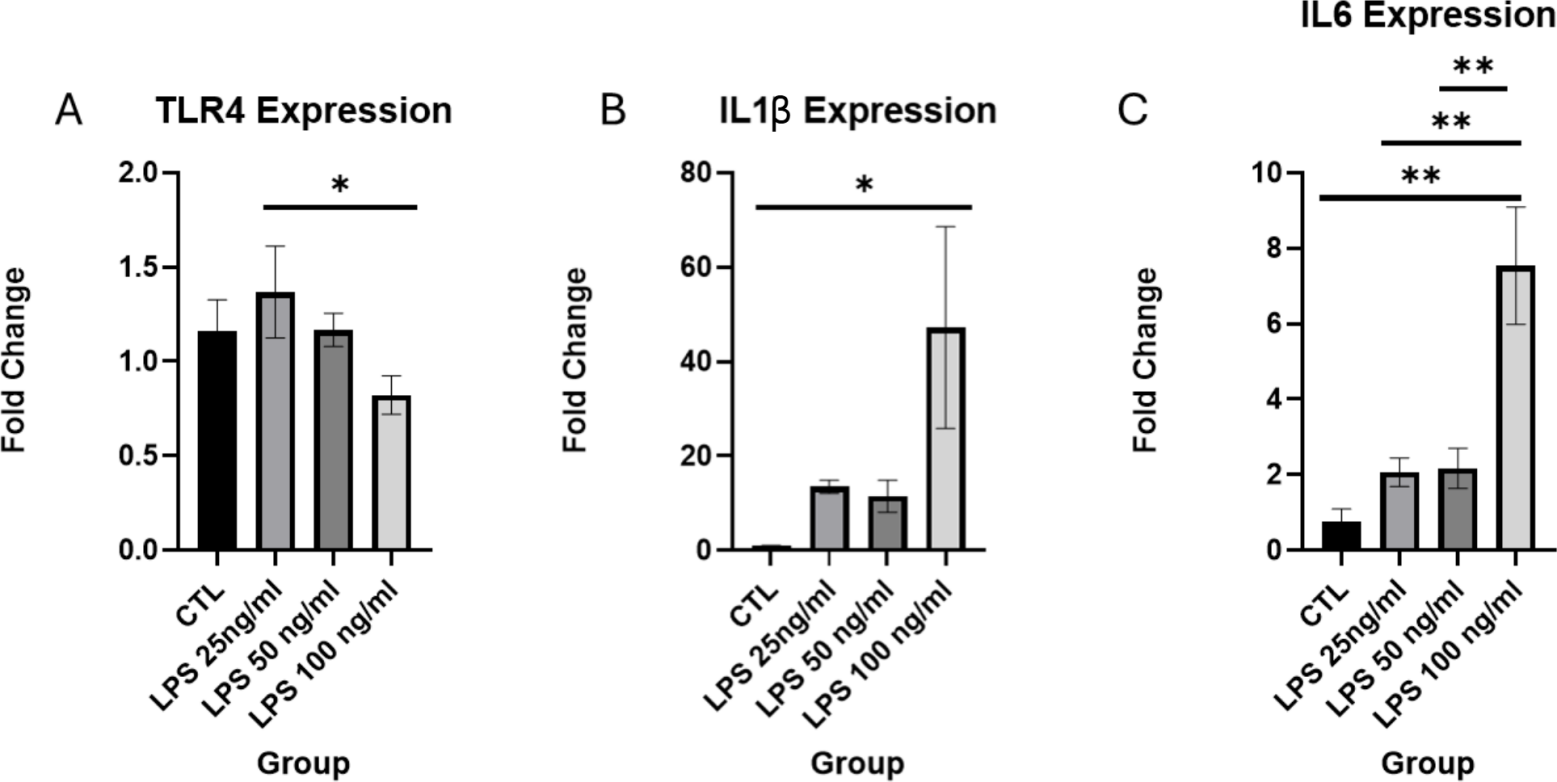
Relative expression of LPS signal transduction related genes: **(A)** TLR4, **(B)** IL1β, **(C)** IL6 expression. *p< 0.05, **p< 0.01.

To directly identify the response of chondrocytes to the inflammatory insult in a system that is devoid of any immune cells and surrounding bone tissue, we proceeded to *in vitro* studies in a cell culture of ATDC5 cells. As one of the major pathways identified by DAVID analysis was “response to LPS”, we added LPS to the culture medium, to represent the inflammatory environment to which the EGP was exposed to *in vivo.* First, undifferentiated cells were exposed to LPS (25, 50, and 100 ng/ml) for 24 hours to evaluate their viability and responsiveness to LPS. The expression of Toll-Like Receptor 4 (TLR4) was examined and confirmed, and exposure to 100 ng/ml LPS for 24 hours resulted in a significant upregulation of the proinflammatory cytokines interleukin (IL)-1β and IL-6 (**Figure 8**).

**Figure 9:**
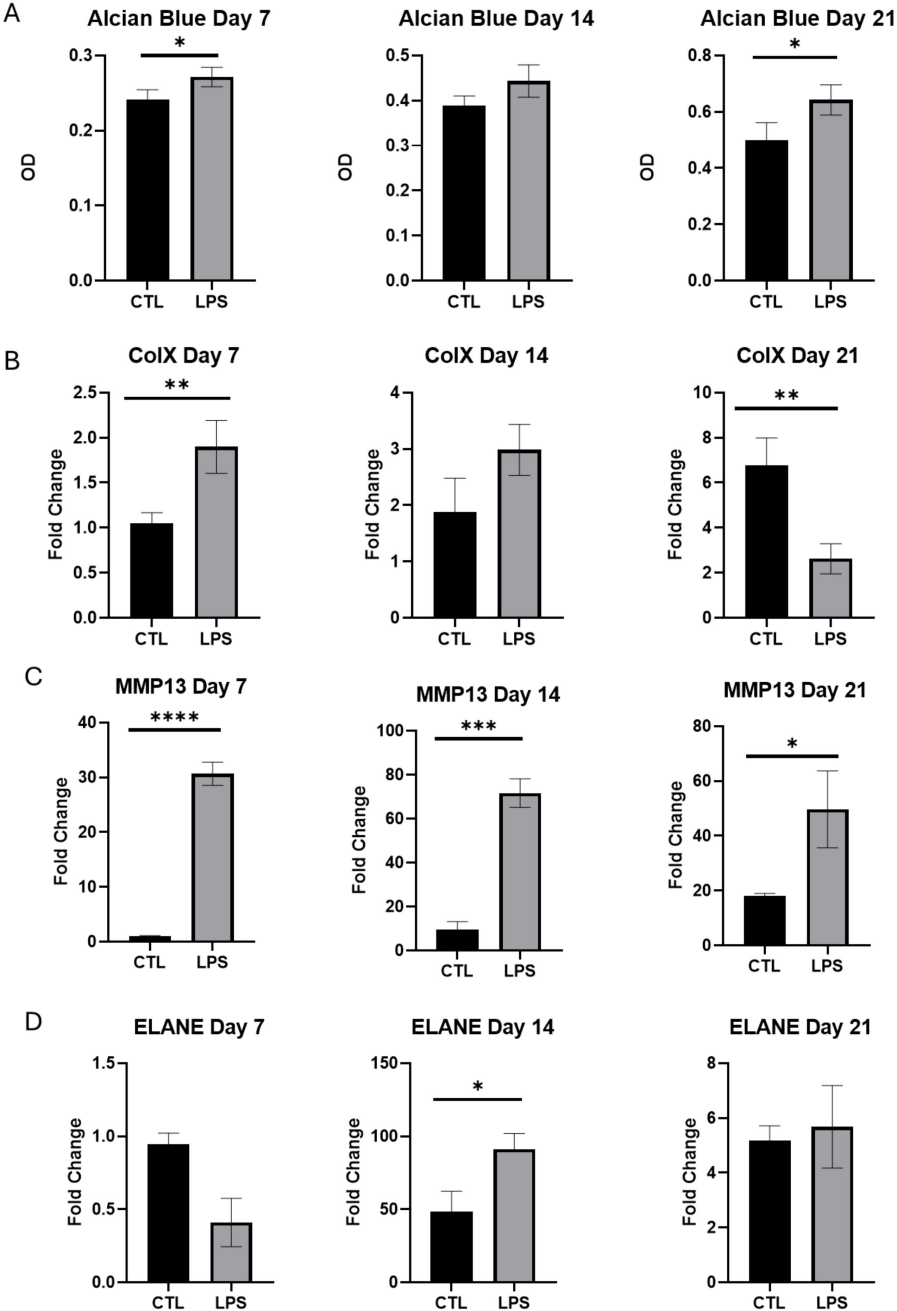
**(A)** Alcian Blue staining OD in ATDC5 cells during differentiation days 7,4,21, **(B)** ColX relative expression during differentiation days 7,4,21, **(C)** MMP13 relative expression during differentiation days 7,4,21, **(D)** ELANE relative expression during differentiation days 7,4,21. *p< 0.05, **p< 0.01, ***p< 0.001, ****p< 0.0001.

Next, we investigated whether ATDC5 express Elane, Ctsg and MPO. On days 14 and 21 of differentiation, ATDC5 cells express ELANE while, Ctsg and MPO were not expressed at any time point (whereas both genes were robustly expressed in spleen samples, which served as a technical control). Interestingly, exposure of the cells to LPS significantly increased the expression level of Elane on day 14 (by 1.5-fold), with a tendency to increase on day 21. These findings confirm that ATDC5 cells respond to LPS through an active TLR4 receptor throughout all differentiation stages (**Figure 9**).

AB staining was used to monitor the increased secretion of proteoglycans, sialomucins, and sulfomucins^18^ as an indicator of chondrogenic differentiation at days 7, 14, and 21. As anticipated, AB levels increased with differentiation in the control group. Interestingly, LPS-treated cells showed a pronounced enhancement in AB staining relative to untreated controls (p<0.05 on days 7 and 21 and p=0.05 on day 14).

To further investigate the effect of LPS on cell differentiation we checked the level of expression of Collagen type X (Col X) and metalloproteinase 13 (MMP13), known to be characteristic of the hypertrophic zone, at all 3 time points (7, 14 and 21d). Indeed, there was a progressive increase in the expression level of Col X in the control cells with differentiation; while exposure to LPS resulted in a premature significant upregulation of Col X expression on day 7 compared to the control (**Figure 9**). On day 21, LPS treated cells showed significant downregulation of Col X. The level of expression of the other differentiation marker, MMP13, also progressively increased with differentiation in the control cells. Again, LPS treatment prematurely increased expression levels from day 7 onwards (by 30 fold, 15 fold and 2.5 fold in days 7, 14 and 21 respectively) (**Figure 9**).

## 4 Discussion

The most important finding of this work is the identification of ELANE in chondrocytes of the EGP and its involvement in growth attenuation during DSS induced colitis. Our study proposes a novel mechanism, involving the activation of ELANE, a serine proteinase previously shown to be secreted by neutrophils in response to inflammatory stimuli (18, 19) that we found to be expressed and upregulated in the EGPs of rats treated with DSS. To the best of our knowledge, this is the first report showing that EGP chondrocytes express ELANE, and specifically its upregulation in both the EGP in DSS induced colitis and in ATDC cells exposed to LPS. We hypothesize that ELANE, together with MMP13, is upregulated by inflammatory stimuli, leading to ECM degradation and, consequently, impaired EGP growth. These findings offer a potential, complementing mechanism through which IBD impairs skeletal growth. The role of ELANE in cartilage degradation and bone re-modelling has been well-established in rheumatoid arthritis and osteoarthritis (20) (OA), but its expression in chondrocytes of the EGP and its involvement in growth attenuation during IBD is a novel contribution of this study.

IBD is a group of chronic disorders causing inflammation of the digestive tract, leading to symptoms such as abdominal pain, weight loss, and malabsorption. Nutritional deficiencies and elevated inflammatory cytokines contribute to growth impairment in pediatric patients (1). IBD impairs growth by affecting both the GH/IGF-1 axis and the EGP. Inflammatory cytokines such as IL-1β, IL-6, and TNF-α induce GH resistance by upregulating SOCS3, which inhibits GH receptor signaling via the JAK2–STAT5 pathway and promotes receptor degradation. They also disrupt IGF binding proteins, reducing IGF-1 bioavailability (21–23). IL-6 primarily affects growth through the GH/IGF-1 axis, while IL-1β and TNF-α impair chondrocyte proliferation (24, 25).

DSS induced colitis caused decreased weight in the treated group with a significantly lower food consumption and shorter colons. EGPs were significantly shorter in DSS treated rats, showing a proportionate reduction in both the proliferative and hypertrophic zones.

Enrichment of terms related to cell cycle checkpoint regulation, mitotic spindle organization, and DNA replication aligns with the upregulation of both G2M checkpoint and E2F target pathways seen in the GSEA. These findings may suggest that EGP cells exposed to DSS may be experiencing replication stress or DNA damage, prompting engagement of repair mechanisms and cell cycle regulation, in accordance with the reduction in the EGP total height. The G2/M checkpoint signaling pathway typically delays mitosis in response to DNA damage or replication stress and its activation indicates that cell cycle may be arrested (26). Concurrently, the E2F targets pathway is generally associated with conditions that favor cell proliferation and facilitate progression from the G1 to S phase (27). The simultaneous activation of proliferation-associated pathways without corresponding proliferation (as seen in our study by decrease in cell count in EGP in the HZ) has been previously observed in contexts where E2F activity is elevated but downstream checkpoint control is impaired (28), a pattern consistent with our findings. Additionally, GO term analysis further supports the presence of DNA damage and replication stress, as most enriched terms are related to DNA repair, chromatin remodeling, and catalytic activity acting on DNA. These transcriptomic changes are consistent with our histological observations. In DSS-treated rats, the total height of the EGP was reduced, and the number of cells in the hypertrophic zone (HZ) was significantly decreased, although cell size remained unchanged. These findings align with previous reports that DSS-induced colitis triggers DNA damage (29) response and is associated with suppressed proliferation within the EGP.

The three DE genes ELANE, MPO and Ctsg, are stored in neutrophil azurophilic granules (18, 30) and released through degranulation in response to various inflammatory stimuli (31). In inflammatory diseases of cartilage such as rheumatoid arthritis (RA) and osteoarthritis (OA), roles for these proteinases have been particularly well described as they play a central role in disease etiology. In OA, their release to the ECM was shown to cause tissue damage and destruction of articular cartilage and in RA, infiltration of neutrophils and neutrophil elastase into the synovial fluid correlate with disease severity and tissues degradation (32, 33).

Indeed, ELANE levels are significantly higher in inflammatory arthropathies (32) and ELANE inhibition has also proven effective at reducing articular cartilage destruction in mice (34). Moreover, double knockout mice for ELANE and Ctsg demonstrated a reduced arthritis score compared to wild-type mice (35), which further demonstrates the role of these enzymes in disease progression. In the current study we show, for the first time, that a similar mechanism, involving ELANE, is active in the EGP of young growing rats exposed to DSS induced inflammation.

*In vitro* studies in ATDC5 cell culture enabled us to further investigate the effect of inflammation on chondrocytes and corroborate the results from the RNA seq. LPS was used in ATDC5 cell instead of DSS as two of the pathways connecting the expression of these genes were “response to LPS” and “innate immunity” which were upregulated in the DSS treated EGPs. Furthermore, in pediatric IBD patients, even during remission, there is a significantly higher circulating serum LPS levels (36), making this model further clinically relevant.

ATDC5 cells were selected for their well-established capacity to undergo chondrogenic differentiation in culture. To monitor this process, we assessed the expression of key marker genes characteristic of distinct differentiation stages. In particular, ColX and MMP13 are commonly used as markers of the hypertrophic zone (typically days 21–28 in culture). Following LPS exposure, this normal differentiation pattern was disrupted: ColX expression rose prematurely and then rapidly declined, indicating early hypertrophy, while MMP13 expression increased early and remained persistently elevated, reflecting sustained ECM-degrading activity. Together, these findings suggest a dysregulated differentiation process, in which matrix degradation begins before chondrocytes reach full maturation.

Gene expression in ATDC5 cells identified upregulation of Elane on day 14, which corresponds with the pre-hypertrophic area. This finding is of paramount importance as neutrophil elastase were shown to prime MMP13 (20, 37) that subsequently cleave both Col II and Col X present in the PZ (above) and the HZ (below) respectively. This may suggest that Elane affects both areas of the EGP, leading to the degradation of both types of collagens thus proportionally decreasing EGP height. MPO and Ctsg were not expressed by ATDC5 in any of the differentiation days, suggesting that they were from the surrounding bone tissue.

ATDC5 cells typically exhibit ECM deposition as they differentiate, with increased AB staining intensity serving as an indicator of more advanced differentiation. In our study, we surprisingly observed premature AB staining in the LPS treated cells, as opposed to the control cells. While AB staining is typically used to follow the differentiation of ATDC5 cells, we unveiled a phenomenon where LPS treated cells exhibited a higher AB staining. However, as mentioned before, qPCR analysis of ColX and MMP13 expression revealed a divergent pattern in LPS-treated cells compared to the expected differentiation trajectory of ATDC5 cells. Col X expression peaked early and declined significantly by day 21, suggesting premature differentiation and cellular senescence (38). MMP13 levels also exhibited an early significant increase on day 7 (by 40-fold), which stayed significant throughout the entire differentiation. The discrepancy between the behavior of AB level and expression level maybe attributed to the significant increase in ELANE expression and the premature ECM degradation in response to inflammatory insults. Indeed, in some cases of OA, AB staining was found to be stronger in inflamed menisci when compared to healthy controls (39, 40) this may indicate enhanced degradation of ECM in the cartilage, leading to more available areas that can be stained by AB.

Our study proposes a novel mechanism by which chronic inflammation may impair skeletal growth. We demonstrate that chondrocytes within the EGP express ELANE in response to inflammatory stimuli of different sources, independent of immune cell infiltration, as we recreated the inflammatory environment in a cell culture. This intrinsic expression of ELANE along with the upregulation MMP13 suggests a direct catabolic effect on the ECM, as observed by AB staining of these cells. ELANE was shown to prime and activate MMP13, both in arthritic cartilage and cell cultures (20), the increase in the level of both genes, hence, strongly suggests activation of MMP13.

These findings reveal a previously unrecognized pathway by which systemic inflammation can impact EGP physiology. Understanding the mechanisms underlying EGP dysfunction in IBD is essential for developing targeted treatments for IBD-related growth attenuation. ELANE inhibitors, currently under investigation as potential therapies for osteoarthritis and rheumatoid arthritis (40), help preserve cartilage by limiting MMP activity. Given the suggested role of ELANE in colitis-induced growth attenuation observed in this study, future incorporating ELANE inhibitors into treatment strategies for children with inflammation-related growth failure may be a promising novel approach. Further studies are needed to evaluate the therapeutic potential of targeting ELANE and related proteases to alleviate growth impairment in pediatric IBD.

We also acknowledge that the manuscript has some limitations: 1) While Ctsg and MPO were detected by RNA seq of the EGP, they were not expressed by ATDC5 cells. This may be due to residual bone contamination as RNA was extracted manually from the EGP. Laser capture fractionation could not be performed due to the age of the animals. 2) In this study, only males were tested. This was because males enter puberty later enabling a longer period of intervention (41), in the next studies, the effect on females should be added.

## Data Availability

RNA Seq data was uploaded to https://www.ebi.ac.uk/biostudies/arrayexpress/studies/E-MTAB-16246 accession number- E-MTAB-16246.

## Supplementary material

Supplementary Figure 1 STRING analysis of DE genes from RNA Seq results. Arrow pointing to strongest possible connections.

Supplementary Figure 2 DAVID bioinformatic analysis of DE genes from RNA Seq results.
