# Supplementary figures and images for: Neutrophil elastase (Elane) may serve as a potential therapeutic target in inflammatory bowel disease-associated growth attenuation: focus on the epiphyseal growth plate in young male rats
Source: Front Endocrinol (Lausanne). 2025 Dec 2;16:1688220. doi: 10.3389/fendo.2025.1688220 (PMC12705387; doi:10.3389/fendo.2025.1688220)

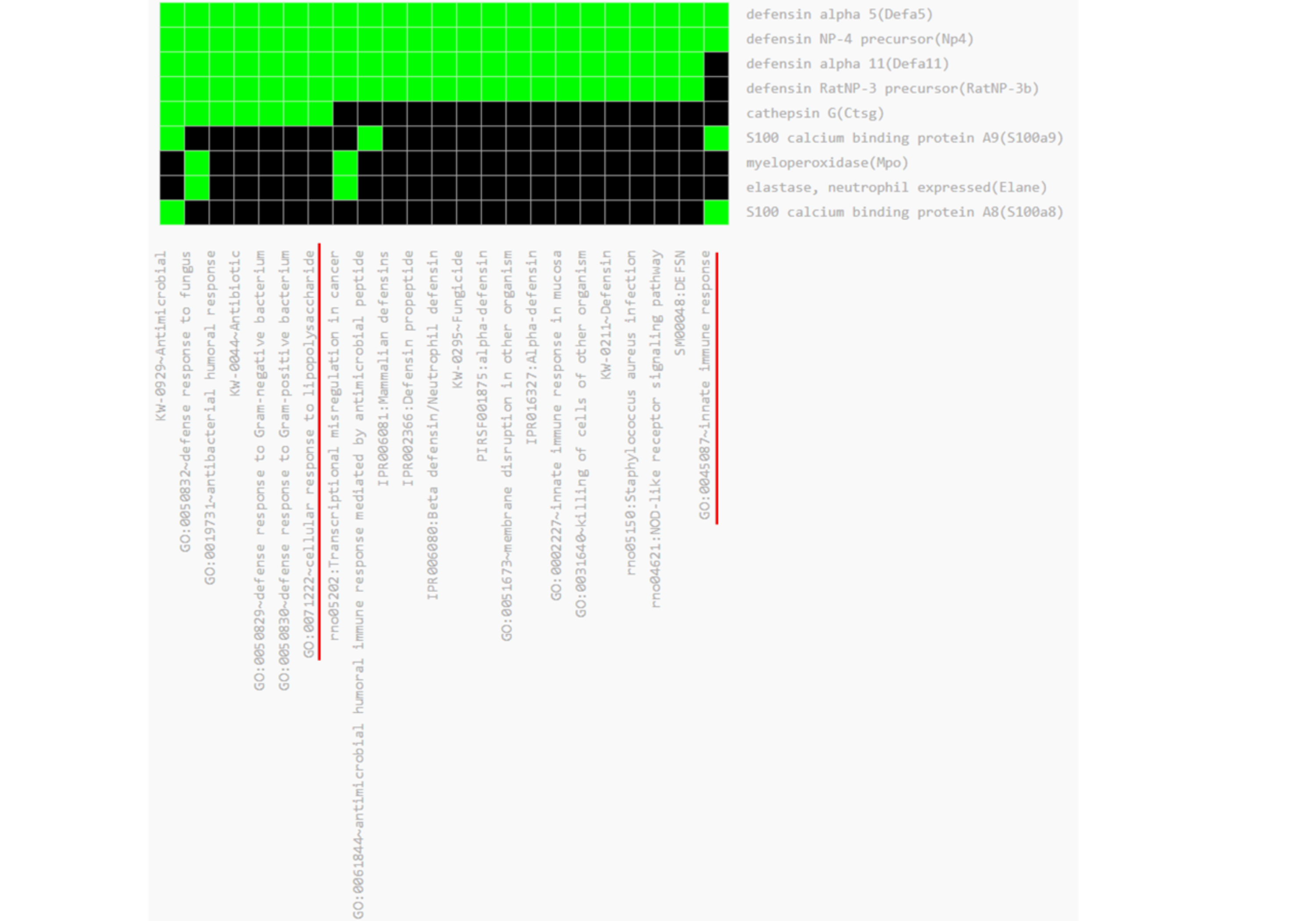

Supplement: Supplementary Figure 1 — STRING analysis of DE genes from RNA Seq results. Arrow pointing to strongest possible connections. [file Image1.jpeg]

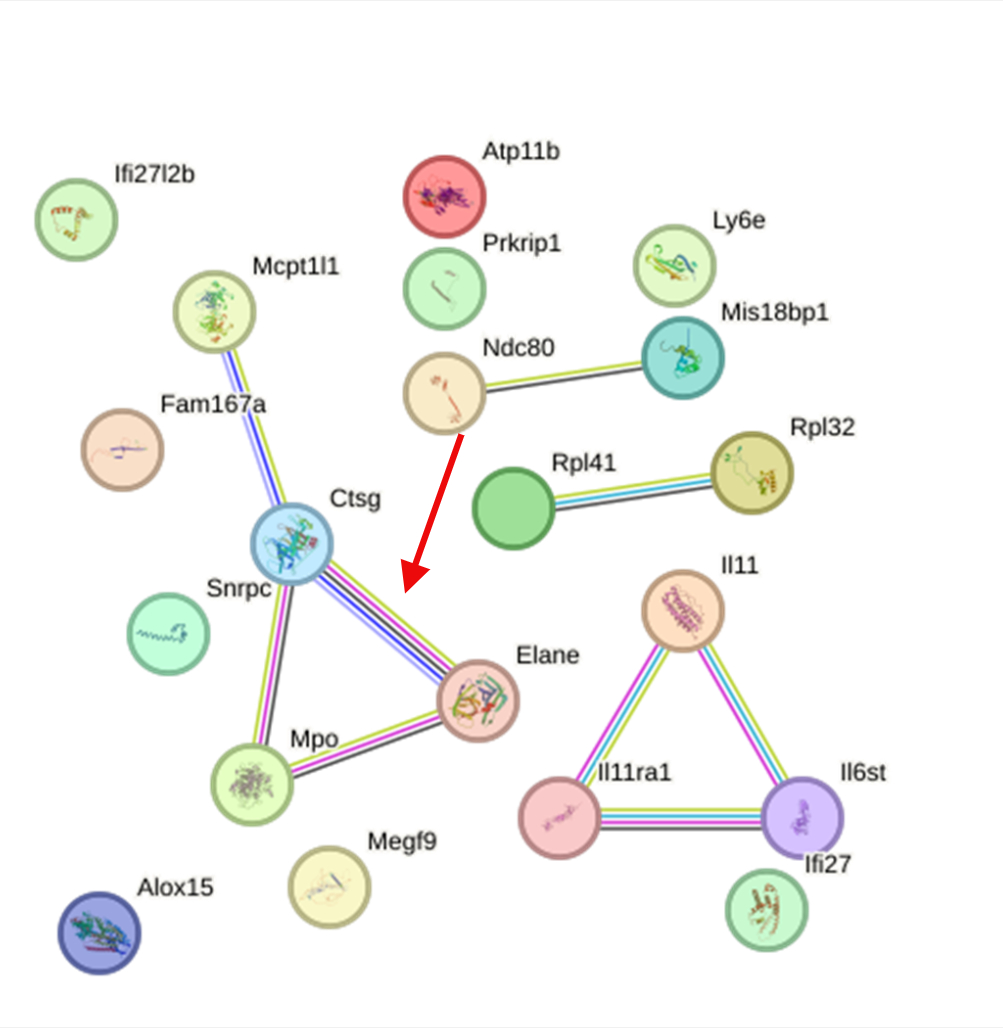

Supplement: Supplementary Figure 2 — DAVID bioinformatic analysis of DE genes from RNA Seq results. [file Image2.jpeg]
